# Transient dynamics of infection transmission in a simulated intensive care unit

**DOI:** 10.1371/journal.pone.0260580

**Published:** 2022-02-03

**Authors:** Katelin C. Jackson, Christopher T. Short, Kellan R. Toman, Matthew S. Mietchen, Eric Lofgren, for the CDC MInD-Healthcare Program

**Affiliations:** 1 Paul G. Allen School for Global Animal Health, Washington State University, Pullman, WA, United States of America; 2 Dept. of Mathematics, Washington State University, Pullman, WA, United States of America; Nanyang Technological University, SINGAPORE

## Abstract

Healthcare-associated infections (HAIs) remain a serious public health problem. In previous work, two models of an intensive care unit (ICU) showed that differing population structures had markedly different rates of *Staphylococcus aureus* (MRSA) transmission. One explanation for this difference is the models having differing long-term equilbrium dynamics, resulting from different basic reproductive numbers, *R*_0_. We find in this system however that this is not the case, and that both models had the same value for *R*_0_. Instead, short-term, transient dynamics, characterizing a series of small, self-limiting outbreaks caused by pathogen reintroduction were responsible for the differences. These results show the importance of these short-term factors for disease systems where reintroduction events are frequent, even if they are below the epidemic threshold. Further, we examine how subtle changes in how a hospital is organized—or how a model assumes a hospital is organized—in terms of the admission of new patients may impact transmission rates. This has implications for both novel pathogens introduced into ICUs, such as Ebola, MERS or COVID-19, as well as existing healthcare-associated infections such as carbapenem-resistant Enterobacteriaceae.

## Introduction

Healthcare-associated infections are a serious source of morbidity and mortality, and are likely to continue to be so as rates of antibiotic resistance increase. In addition to their health-related complications, these infections are also a significant burden on the resources of the healthcare system. In 2015, the Department of Health and Human Services’ Hospital-Acquired Condition Reduction Program (HACRP) levied approximately $330 million in penalties against hospitals with high infection rates [1]. For both reasons, reducing HAIs is a top priority for healthcare safety and quality teams.

One such HAI, for which there has been some success in reducing rates, is methicillin-resistant *Staphylococcus aureus* (MRSA). MRSA is especially difficult to treat and can be very dangerous to immune-compromised individuals and other vulnerable patients such as those in the intensive care unit (ICU) or a burn ward [2]. MRSA is most often treated with vancomycin, a drug with a myriad of potential side effects, and a treatment failure rate of nearly 50% [3]. Because of the difficulty in treating patients with MRSA once they have developed a clinical infection, a great deal of time and attention is placed on the prevention of the initial colonization of a patient with the bacteria, involving interventions such as hand hygiene or contact precautions.

One tool to study HAI transmission and develop new interventions is the use of mathematical models, the most frequent of which in hospital epidemiology are based off of the Ross-Macdonald model. Ross and Macdonald developed the theory of transmission dynamics and control for mosquito transmitted pathogens. Since 1899, this has been expanded on by a number of others, including Waite [4], Lotka [5], Sharpe [5], Dietz [5], Bailey [5], Koella [6], and others. These extensions are now widely regarded as a set of models defined by a simplified set of assumptions regarding transmission—primarily that transmission of a pathogen takes place between two or more classes (i.e. humans and mosquitos) rather than within a single class. Given the importance of hand hygiene and environmental contamination in healthcare-associated transmissions, and the relatively low mobility of patients in intensive care, these models are readily adapted to the healthcare environment.

In a previous work Mietchen *et al*., 2019 [7] explored three models build on this framework, considering different methods of representing the population structure of an ICU. These were: 1) treating all patients as a single well-mixed group with nurses and doctors combined into a single staff type,(SST)—most analogous to a classic formulation of a Ross-Macdonald model, 2) breaking nurses and doctors into two staff types with type-specific contact parameters while maintaining the well-mixed structure,(Nurse-MD), and 3) representing the ICU as a meta-population, where patients are divided into groups of three with a single attending nurse per group, while the doctor sees all patients,(meta-population). These models were chosen to represent a continuum of compartmental model complexity, from the simple and relatively analytically tractable SST model to the meta-population model, which is considerably more complex in representational form, and represents something of an intermediate step between compartmental models and network models with granular representations of patient and provider contact.

It was shown that the meta-population model had markedly lower infection rates using the same parameterization, and generally was less sensitive to changes in parameter values. An exploration of one parameter in particular, *γ*, the proportion of time spent by a nurse exclusively treating their assigned patients, which may be varied and which allows the meta-population model to reduce to the Nurse-MD model, showed a non-linear relationship between its value and MRSA acquisitions. This study focuses only on this pair of models, because of their relatedness.

This previous work, however, focused primarily on the long-term dynamics of these models, obtained purely by stochastic simulation. Left unexplored was the mathematical explanation for these results. One plausible explanation is that, despite having very similar formulations, each model had a different basic reproductive number, *R*_0_, which determines whether an epidemic will die out (*R*_0_ < 1), stay in a constant state of equilibrium (*R*_0_ = 1) or continue to grow (*R*_0_ > 1). An alternative hypothesis is that these differences in simulated infection rates are driven not by the model’s long-term equilibrium states, but with shorter-term phenomena known as transient dynamics.

We define transient dynamics as non-permanent, short-term behaviors of the model, driven by stochasticity, small perturbations (such as the admission of a colonized patient), etc. Effectively, the behavior of the model when it is expressly not at equilibrium. In this paper we explore the transient dynamics of infection transmission in these systems, to demonstrate that the two models, Nurse-MD and meta-population, have the same *R*_0_ and that the transient dynamics of the these models are what drive a series of small outbreaks rather than the long-term dynamics. To illustrate this, we examine the model’s sensitivity to relatively subtle, but realistic, differences in starting conditions.

## Methods

### Intensive care unit model

We consider a 18-bed, single occupancy ICU, where patients are assumed, due to their critical status, to be immobile. As MRSA is not airborne, this then restricts the available MRSA transmission pathways to strictly healthcare worker (HCW) mediated patient-to-patient transmission. The role of environmental contamination is represented by modeling the contact rate in terms of “direct care tasks”, which involve a healthcare worker touching either a patient or their surrounding environment, rather than in terms of patient body-contacts alone.

**Fig 1:**
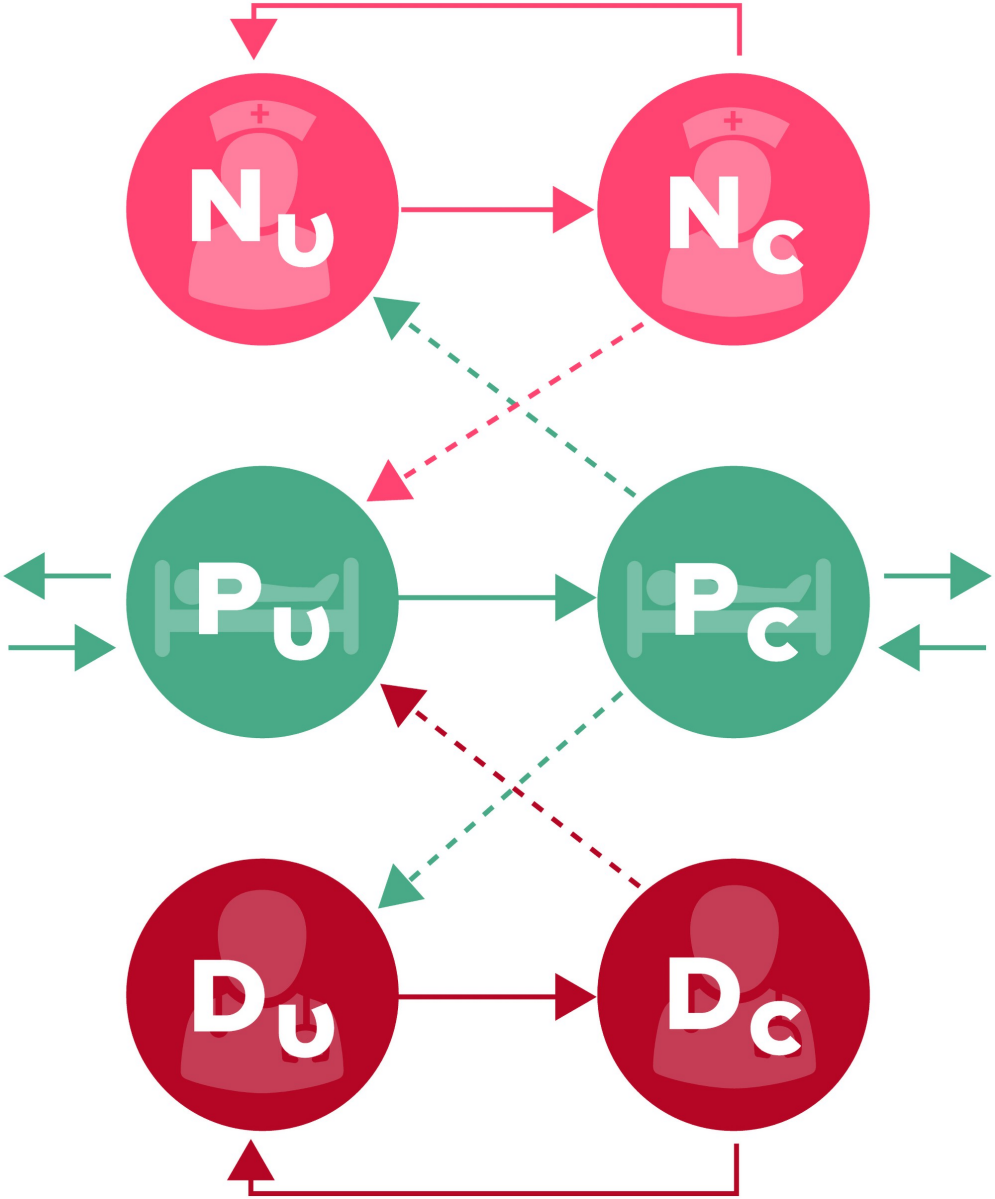
Schematic representation of the compartmental flow of a mathematical model of methicillin-resistant *Staphylococcus aureus* (MRSA) acquisition with nurses and intensivists separated into different staff types. Solid arrows indicate possible transition states, while dashed arrows indicate potential routes of MRSA contamination or colonization. Nurses and doctors are classified as uncontaminated (*N*_*U*_ or *D*_*U*_) and contaminated (*N*_*C*_ and *D*_*C*_), while patients are classified as uncolonized (*P*_*U*_) or colonized (*P*_*C*_). Figure by Eric Lofgren is licensed under CC BY 4.0.

**Fig 2:**
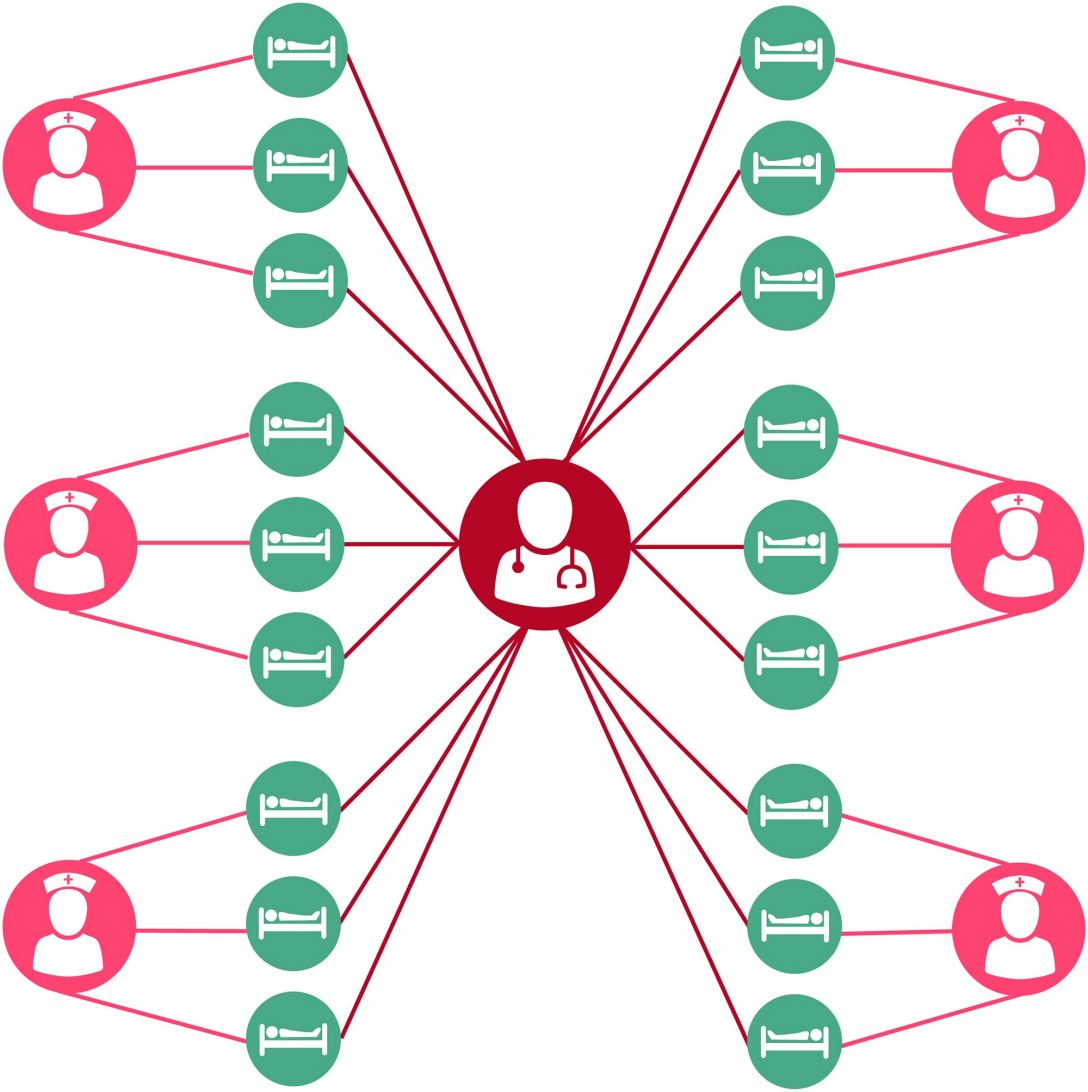
Schematic representation of a meta-population model of methicillin-resistant *Staphylococcus aureus* (MRSA) acquisition. Patients (blue) are treated by a single assigned nurse (orange). A single intensivist (red) randomly treats all patients. Figure by Eric Lofgren is licensed under CC BY 4.0.

As previously described, two variations for our model ICU are analyzed. In first model (Fig 1), the 18 patients are still viewed as a group, while the six nurses and single doctor are treated as separate types of individuals, with role-specific parameters for their contact rate. This formulation assumes random mixing where all healthcare workers care for all patients in equal amounts, and is a simple representation of an ICU of a form commonly used either for computational efficiency in a larger model, or for analytical tractability. In the second model, we represent the ICU as a meta-population (Fig 2), wherein the patients are no longer lumped together but instead placed in groups of three with a single nurse attending each group while the doctor sees all patients. An important feature of this model is the inclusion of a parameter, *γ*, which represents the proportion of time a nurse spends with their assigned patient group. These assignments can be thought of as representing a number of different policies or procedures, such as continuity of care policies, where healthcare workers are repeatedly assigned the same patients to ensure consistent care, or a hospital build environment that naturally separates patients into groups due to architectural structure. Departures from these assignments can be thought of as difficult procedures requiring multiple healthcare workers, cross-coverage during breaks, etc. When *γ* = 1 this can be considered a strict assignment, whereas the system is equivalent to a mass action model when $\gamma =\frac{1}{C}$, where C is the number of patient groups. The value of this parameter has been shown in previous simulation work [7] to non-linearly decrease the number of incident acquisitions of MRSA within the ICU with increasing values of *γ*. This formulation also reflects many of the realities of staffing, the desire for continuity of care between healthcare providers, and even the hospital built environment, where the placement of patient beds, nursing stations, etc. creates logical groupings. A table containing the parameter values is below in Table 1.

**Table 1:** Parameter values for models of MRSA transmission in an ICU.

Name	Value	Interpretation	Source(s)
*ρ*_*N*_	3.973	Nurse direct care tasks per hour	[9, 10]
*ρ*_*D*_	0.181	Doctor direct care tasks per hour	[9, 10]
*σ*	0.054	Hand contamination probability	[11]
*ψ*	0.029	Probability of patient colonization given contaminated contact	Fitted to [12, 13]
*θ*	0.00949	Probability of discharge	[13]
*ν*_*C*_	0.0779	Proportion of admissions colonized with MRSA	[13]
*ν*_*U*_	(1-*ν*_*C*_)	Proportion of uncolonized admissions	[13]
*ι*_*N*_	6.404	Nurse hand washing rate—11.02 nurse tasks per hour with 56.55% compliance and 95% efficacy	[9, 10, 13, 14]
*ι*_*D*_	1.748	Doctor hand washing rate—3.25 doctor tasks per hour with 56.55% compliance and 95% efficacy	[9, 10, 13, 14]
*τ*_*N*_	2.728	3.30 nurse gown/glove changes per hour with 82.66% compliance	[11, 13, 15]
*τ*_*D*_	0.744	0.90 doctor gown/glove changes per hour with 82.66% compliance	[11, 13, 15]
*μ*	0.002083	Natural decolonization rate median 20 days	[16]
DT	1	Total Number of Doctors	
NT	6	Total Number of Nurses	
PT	18	Total Number of Patients	
HWT	7	Total Number of Health Care Workers	
NPT	1	Total Number of Nurses per ‘cohort’	
PPT	3	Total Number of Patients per ‘cohort’	

For each of these models, we consider the ICU to always be at capacity as a discharge will immediately lead to an admission, maintaining a steady-state population [8]. Further detail on the construction, implementation and parameterization of the models may be found in [7]. The equations governing each of the two models is below.

### Nurse-doctor model

$$\begin{array}{c} \frac{dP_{U}}{dt}=-\rho_{N}\psi P_{U}\frac{N_{C}}{N_{C}+N_{U}}-\rho_{D}\psi P_{U}\frac{D_{C}}{D_{C}+D_{U}}+\theta \nu_{U}P_{C}+\mu P_{C}-\theta \nu_{C}P_{U} \end{array}$$ (1)

$$\begin{array}{c} \frac{dP_{C}}{dt}=\rho_{N}\psi P_{U}\frac{N_{C}}{N_{C}+N_{U}}+\rho_{D}\psi P_{U}\frac{D_{C}}{D_{C}+D_{U}}-\theta \nu_{U}P_{C}-\mu P_{C}+\theta \nu_{C}P_{U} \end{array}$$ (2)

$$\begin{array}{c} \frac{dN_{C}}{dt}=-\iota_{N}N_{C}-\tau_{N}N_{C}\frac{P_{C}}{P_{C}+P_{U}}+\rho_{N}\sigma N_{U}\frac{P_{C}}{P_{C}+P_{U}} \end{array}$$ (3)

$$\begin{array}{c} \frac{dN_{U}}{dt}=\iota_{N}N_{C}+\tau_{N}N_{C}\frac{P_{C}}{P_{C}+P_{U}}-\rho_{N}\sigma N_{U}\frac{P_{C}}{P_{C}+P_{U}} \end{array}$$ (4)

$$\begin{array}{c} \frac{dD_{U}}{dt}=\iota_{D}D_{C}+\tau_{D}D_{C}\frac{P_{C}}{P_{C}+P_{U}}-\rho_{D}\sigma D_{U}\frac{D_{C}}{P_{C}+P_{U}} \end{array}$$ (5)

$$\begin{array}{c} \frac{dD_{C}}{dt}=-\iota_{D}D_{C}-\tau_{D}D_{C}\frac{P_{C}}{P_{C}+P_{U}}+\rho_{D}\sigma D_{U}\frac{D_{C}}{P_{C}+P_{U}} \end{array}$$ (6)

### Meta-population model

$$\begin{array}{c} \frac{dP_{U_{i}}}{dt}=-\rho_{N}\psi \gamma P_{U_{i}}\frac{N_{C_{i}}}{N_{C}+N_{U}}-\rho_{N}\psi \left(1-\gamma \right)P_{U_{i}}(\frac{N_{C}-N_{C_{i}}}{N_{C}+N_{U}})-\rho_{D}\psi P_{U_{i}}\frac{D_{C}}{D_{C}+D_{U}}\\ +\theta \nu_{U}P_{C_{i}}+\mu P_{C_{i}}-\theta \nu_{C}P_{U_{i}} \end{array}$$ (7)

$$\begin{array}{c} \frac{dP_{C_{i}}}{dt}=\rho_{N}\psi \gamma P_{U_{i}}\frac{N_{C_{i}}}{N_{C_{i}}+N_{U_{i}}}+\rho_{N}\psi \left(1-\gamma \right)P_{U_{i}}(\frac{N_{C}-N_{C_{i}}}{N_{C}+N_{U}})+\rho_{D}\psi P_{U_{i}}\frac{D_{C}}{D_{C}+D_{U}}\\ -\theta \nu_{U}P_{C_{i}}-\mu P_{C_{i}}+\theta \nu_{C}P_{U_{i}} \end{array}$$ (8)

$$\begin{array}{c} \frac{dN_{U_{i}}}{dt}=\iota_{N}N_{C_{i}}+\tau_{N}\gamma N_{C_{i}}\frac{P_{C_{i}}}{P_{U_{i}}+P_{C_{i}}}+\tau_{N}\left(1-\gamma \right)N_{C_{i}}(\frac{P_{C}-P_{C_{i}}}{P_{C}+P_{U}})\\ -\rho_{N}\sigma \gamma N_{U_{i}}\frac{P_{C_{i}}}{P_{U_{i}}+P_{C_{i}}}-\rho_{N}\sigma \left(1-\gamma \right)N_{U_{i}}(\frac{P_{C}-P_{C_{i}}}{P_{C}+P_{U}}) \end{array}$$ (9)

$$\begin{array}{c} \frac{dN_{C_{i}}}{dt}=-\iota_{N}N_{C_{i}}-\tau_{N}\gamma N_{C_{i}}\frac{P_{C_{i}}}{P_{U_{i}}+P_{C_{i}}}-\tau_{N}\left(1-\gamma \right)N_{C_{i}}(\frac{P_{C}-P_{C_{i}}}{P_{C}+P_{U}})\\ +\rho_{N}\sigma \gamma N_{U_{i}}\frac{P_{C_{i}}}{P_{U_{i}}+P_{C_{i}}}+\rho_{N}\sigma \left(1-\gamma \right)N_{U_{i}}(\frac{P_{C}-P_{C_{i}}}{P_{C}+P_{U}}) \end{array}$$ (10)

$$\begin{array}{c} \frac{dD_{U}}{dt}=\iota_{D}D_{C}+\tau_{D}D_{C}\frac{P_{C}}{P_{U}+P_{C}}-\rho_{D}\sigma D_{U}\frac{P_{C}}{P_{U}+P_{C}} \end{array}$$ (11)

$$\begin{array}{c} \frac{dD_{C}}{dt}=-\iota_{D}D_{C}-\tau_{D}D_{C}\frac{P_{C}}{P_{U}+P_{C}}+\rho_{D}\sigma D_{U}\frac{P_{C}}{P_{U}+P_{C}} \end{array}$$ (12)

In the above equations, *γ* represents a percentage of time a nurse would spend tending patients not assigned to him/her. If $\gamma =\frac{1}{6}$ the system reduces to the Nurse-MD model, and *γ* = 1 is the full meta-population model where no nurse tends to unassigned patients. Note that the above equations imply a simplifying assumption that the effect of the patients/nurses not assigned to group *i* can be represented by the sum of all patients/nurses not belonging to group *i*, i.e. $\gamma P_{U_{i}}\sum_{i\neq j}\frac{N_{C_{i}}}{N_{C_{i}}+N_{U_{i}}}=\gamma P_{U_{i}}(\frac{N_{C}-N_{C_{i}}}{N_{C}+N_{U}})$. Here, $N_{C_{i}}$ represents the number of contaminated nurses in group *i* whereas *N*_*C*_ represents the total number of contaminated nurses in the hospital.

### Derivation of *R*_0_

In order to ascertain whether the differing model types under consideration are the result of differing values of *R*_0_, and thus examine whether (or to what extent) the differences in the simulations found in [7] can be explained by differing long-term equilibrium dynamics, we derived both the numerical and symbolic forms for *R*_0_ in each system using the Next Generation Matrix Method.

The Next Generation Matrix, denoted by *K* = F*V^−1^, was used to calculate the basic reproductive number, *R*_0_; it was introduced by Diekmann *et al*. 1990, where they defined *R*_0_ to be the dominate eigenvalue of *K* [17–19]. The Next Generation Matrix is an alternative to the Jacobian method, and is a general method for deriving *R*_0_ in complex compartmental models [20, 21]. Further details, mathematical proofs, and examples can be found in van den Driessche *et al*. 2008 [22], Yang 2014 [19], van den Driessche *et al*. 2017 [21], and O. Diekmann *et al*. 2010 [18].

An important caveat is that this method relies on the notion of a Disease Free Equilibrium, which is rare in hospital epidemiology, where most commonly hospitals are subjected to a continuous influx of new, potentially infective patients. Here, we make the simplifying assumption that the colonization rate of incoming patients, save for the initial colonized patient, is zero. Realistically, this most closely models a nosocomial outbreak of an emerging infectious disease that does not have established transmission in the community, or where the admission of colonized individuals is vanishingly rare.

### Stochastic simulation of meta-population initial conditions

The meta-population model, as it divides the patient population into strictly non-interacting groups, potentially has a starting condition not present in the other models. In any model where $\sum_{i=1}^{n}P_{C,i}\geq 2$, the placement of those patients is potentially relevant. Two foreseeable scenarios were stochastically simulated —one where two patients were attended by the same nurse, and one where each patient was attended by a different nurse. These two conditions were simulated for one year assuming (as with the calculations of *R*_0_) that there were no colonized admissions, and also in a more realistic circumstance where 7.79% of admitted patients were colonized with MRSA, either from the community or elsewhere in the hospital. These simulations were performed using the StochPy package [23] and Python 3.7.

Using a panel of 1,000 runs of each model, we generated Kaplan-Meier survival curves [24] for the time until the first MRSA acquisition and time until the third MRSA acquisition, to assess if there were any differences in the amount or timing of these early initial acquisition events. Statistical significance was assessed using a log-rank test, using the survival package [25] in R 3.5.2. The code and data used in these simulations may be found at http://www.github.com/epimodels/transientdynamics.

## Results and discussion

### Basic reproduction number

The analytical form for *R*_0_ for each of the three models, as well as the specific numerical values for *R*_0_ using the parameters found in Table 1 are shown in Table 2.

**Table 2:** Values of *R*_0_ for two ICU models.

Model	Numerical *R*_0_	Analytic *R*_0_
Nurse-MD	0.2781	$\sigma \psi \frac{\iota_{d}\rho_{n}^{2}+\iota_{n}\rho_{D}^{2})}{\left(\mu +\theta \nu_{u}\right)\iota_{n}\iota_{d}}$
Meta-Population	0.2781	$\psi \sigma \frac{6NPTPPT\iota_{n}\rho_{d}^{2}+PT\iota_{d}\rho_{n}^{2}}{NPT\left(\nu_{u}\theta +\mu \right)\iota_{n}PT\iota_{d}}$

In contrast to the numerical results in Mietchen *et al*., 2019, despite having markedly different simulated rates of infection, the numeric value of *R*_0_ between the Nurse-MD model and the meta-population model is the same, at 0.2781. This value provides two pieces of evidence that the difference in the dynamics of these systems is driven by transient dynamics. The first is that, given this model formulation where contact is based not on individuals, but on clinical care tasks, which are constrained to be equal between the Nurse-MD model and the Meta-Population model, the identical values of *R*_0_ should result in the same long-term equilibrium dynamics. More specifically, with identical values of *R*_0_ less than 1 (given the parameters in Table 1), the long-term equilibrium values of these models should converge to a disease-free equilibrium. Following from this, because both values of *R*_0_ are not only below one, but dramatically below one, absent new introductions of colonized individuals, in both systems MRSA is sub-epidemic and likely to die out. Once colonized individuals are reintroduced into the model, the “long-term” dynamics of MRSA in both systems are, instead, revealed to be a sequential series of short term, stochastically driven and ultimately self-limiting sub-epidemic outbreaks. In such a scenario, short-term transient dynamics inherently predominate.

### Numerical simulation of meta-population initial conditions

**Fig 3:**
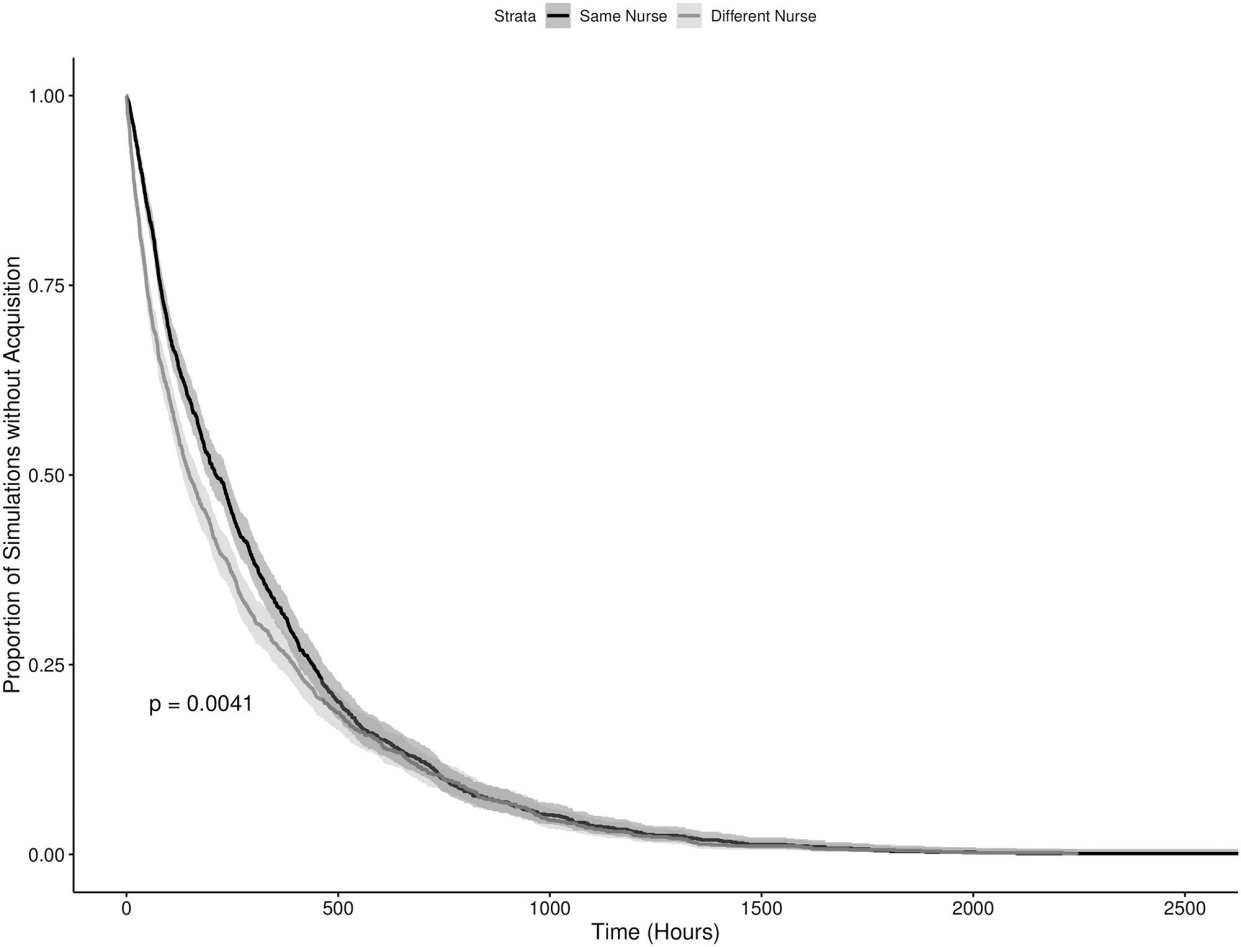
Time to first MRSA acquisition in an ICU meta-population model with potentially colonized admissions. The dark and light grey lines indicate starting conditions where two initially colonized patients are cared for by the same and different nurses respectively, with the shaded regions representing the corresponding 95% confidence intervals.

**Fig 4:**
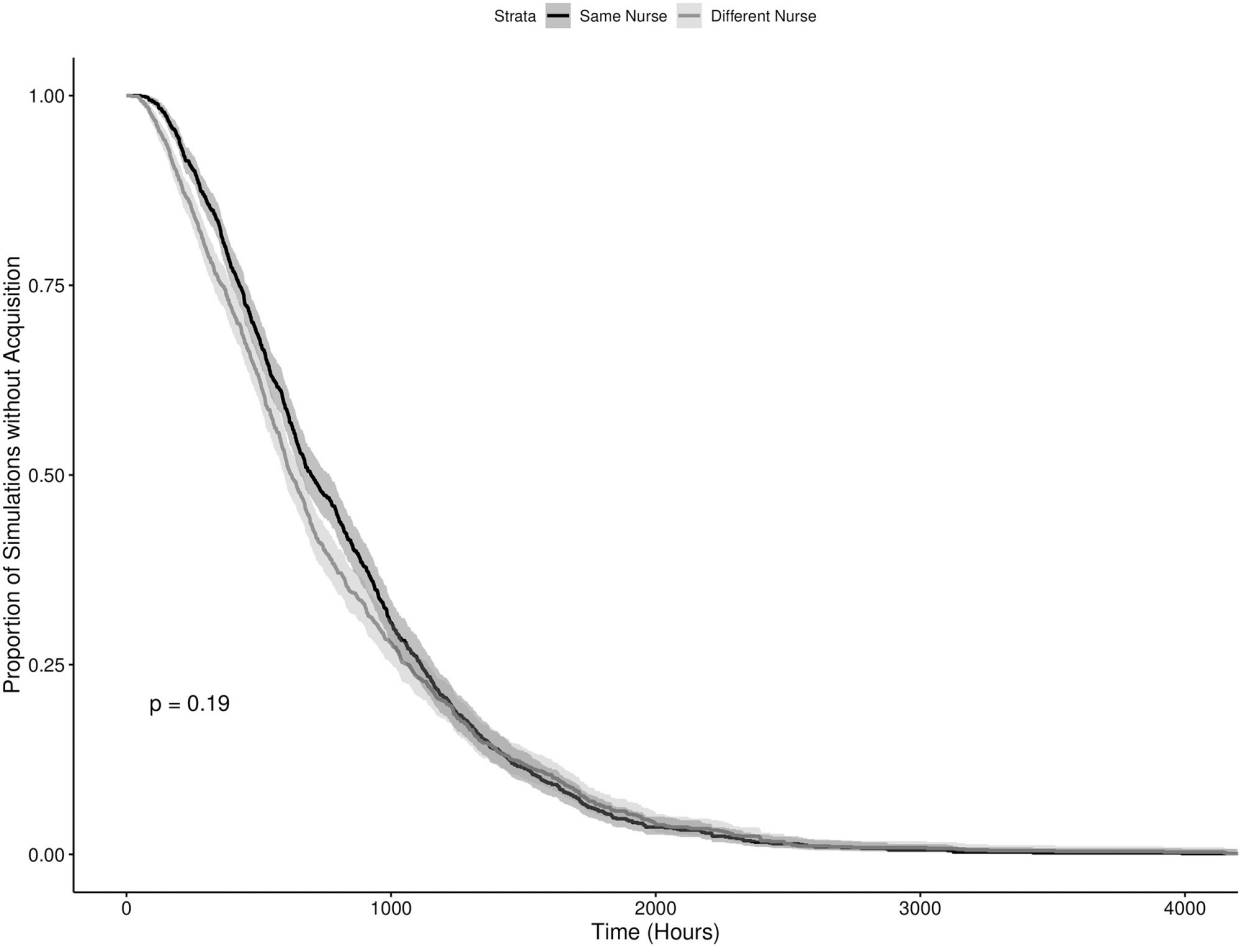
Time to third MRSA acquisition in an ICU meta-population model with potentially colonized admissions. The dark and light grey lines indicate starting conditions where two initially colonized patients are cared for by the same and different nurses respectively, with the shaded regions representing the corresponding 95% confidence intervals.

**Fig 5:**
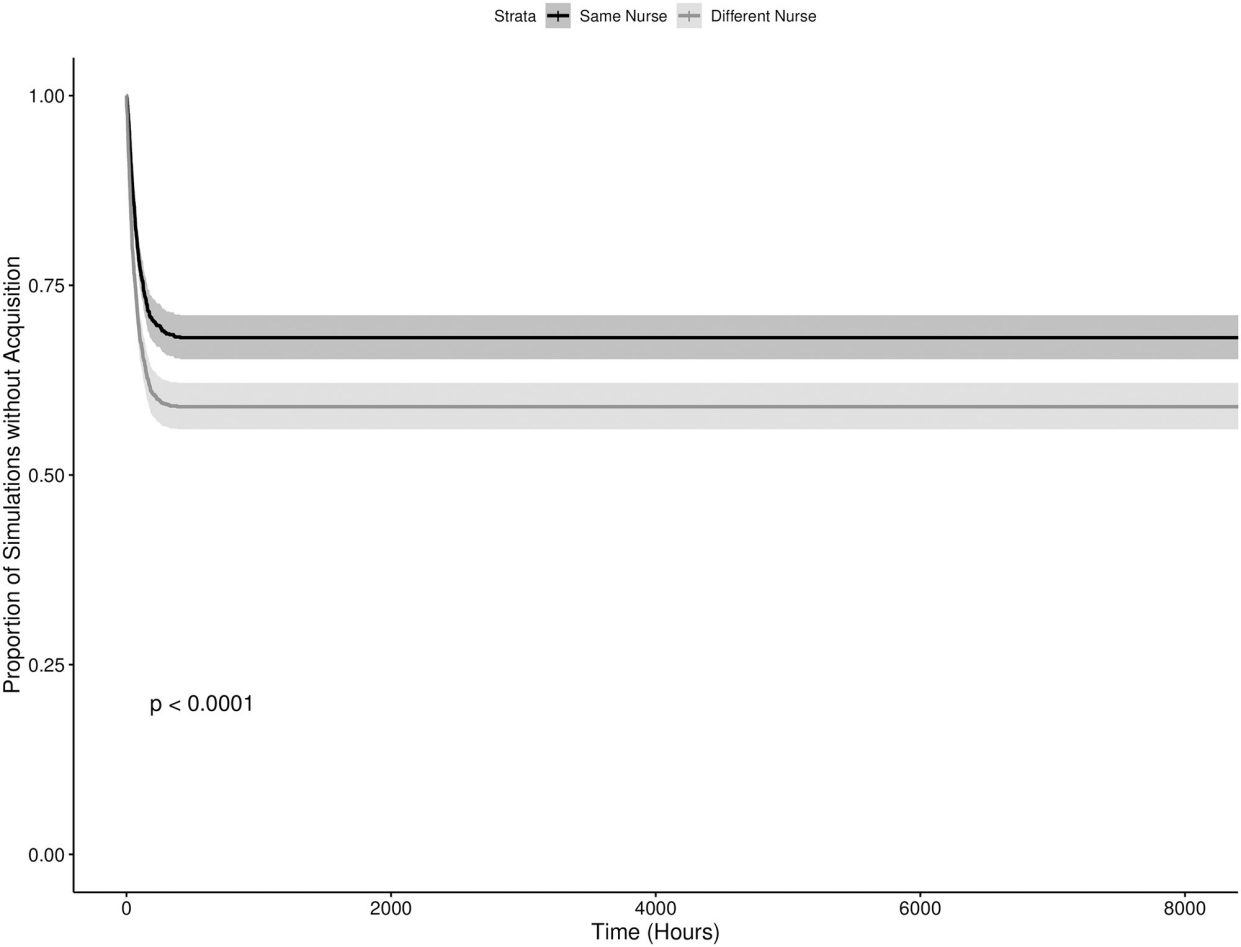
Time to first MRSA acquisition in an ICU meta-population model with no colonized admissions. The dark and light grey lines indicate starting conditions where two initially colonized patients are cared for by the same and different nurses respectively, with the shaded regions representing the corresponding 95% confidence intervals.

**Fig 6:**
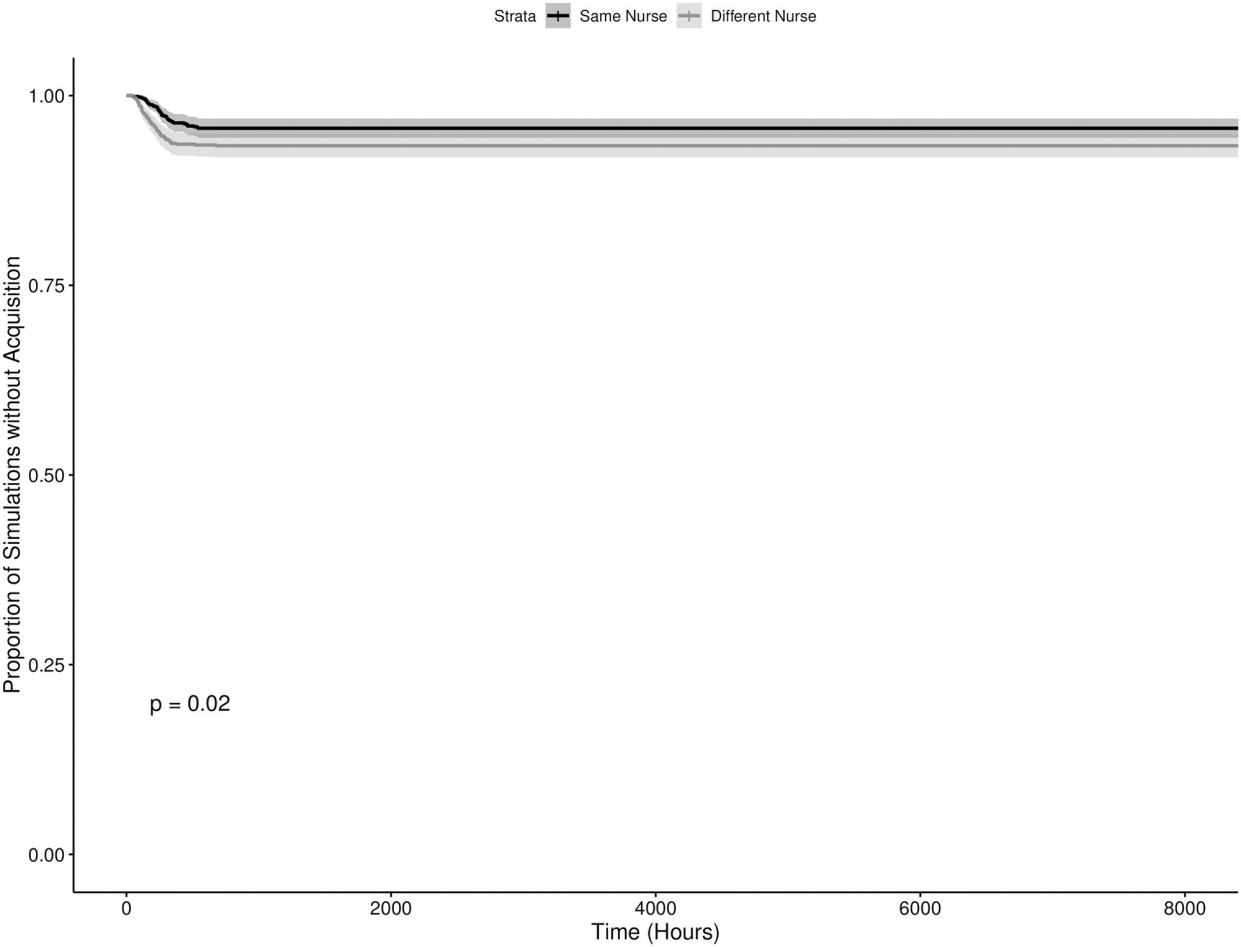
Time to third MRSA acquisition in an ICU meta-population model with no colonized admissionss. Time to Third MRSA Acquisition in an ICU Meta-population Model with No Colonized Admissions. The dark and light grey lines indicate starting conditions where two initially colonized patients are cared for by the same and different nurses respectively, with the shaded regions representing the corresponding 95% confidence intervals.

The results of the numerical simulations can be seen in Figs 3–6, showing the results for the time until the first and third acquisitions of the system with and without colonized admissions respectively. Broadly, there were statistically significant differences in the timing of the first new acquisition in both admission scenarios, with the starting condition where the two “seed” patients were cared for by the same nurse resulting in a faster new acquisition (p = 0.004 and p > 0.0001 in the colonized and uncolonized admission scenarios respectively). This pattern remained significant for the third acquisition in the no colonized admissions scenario (p = 0.02), but not in the colonized admissions scenario. By the fourth acquisition, both starting conditions were statistically indistinguishable within each scenario. Also notably, as the value of *R*_0_ for these models were well below one, the majority of the simulations in the scenario with no further colonized admissions experienced rapid stochastic extinction of the pathogen. Scenarios with the two seed patients treated by different nurses were slightly less likely to go stochastically extinct, with 41.0% of iterations (vs. 31.9%) having a new acquisition within the hospital, and 0.66% iterations (vs. 0.43%) having transmission continue to a third acquisition. These results illustrate the sensitivity of this model to starting conditions, and the dramatic impact of an inherently transient and short-term phenomenon —the admission of a single patient —on the model’s dynamics.

## Conclusion

Our results suggest that, somewhat contrary to most infectious disease systems, the previously simulated differences in MRSA rates depending on how one represents the ICU —or believes that it is organized in reality —is not the result of differing values of *R*_0_ or differences in their long-term dynamics. Indeed, mathematically the Nurse-MD model and the meta-population model are asymptotically the same model, despite the meta-population model having a lower simulated infection rate. Absent the admission of new colonized patients, both models should reach their disease-free equilibrium relatively swiftly.

Instead, we show that the ability of nurses to have a smaller cohort of dedicated patients with which they spend some or all of their time, as well as the differences in starting conditions that arise from how patients are admitted into these cohorts, drives the observed differences, rather than *R*_0_. What is being observed is not two systems reaching different long-term equilibrium states, but rather a series of short-term, stochastic and self-limiting outbreaks that, when viewed over time, begin to appear as persistent, endemic transmission of the pathogen at differing rates. It is the constant re-introduction of disease (via colonized admissions), that keep the models from reaching the disease-free equilibrium implied by their *R*_0_.

This poses a somewhat unusual question for the control of healthcare-associated infections. Unlike most diseases (i.e. COVID-19), *R*_0_ has already been driven below one. The transient, ephemeral nature of the dynamics of this system shifts the focus from controlling spread within an ICU as a persistent phenomena to hardening the ICU against introductions of disease, and the subsequent stochastic transmission events that arise from it. Importantly, this mindset is applicable not only to common pathogens such as MRSA or *C. difficile* but also emerging healthcare-associated pathogens such as carbapenem-resistant Enterobacteriaceae, *Candida auris*, or pathogens such as Ebola, MERS or of more recent concern, COVID-19.

Also evident in the results is further support for the practice of placing colonized patients under the care of the same nurse (known as “cohorting”). We can also see through Figs 5 and 6 that barring the onslaught of incoming patients already colonized with MRSA, the idea of grouping colonized patients under the care of the same nurse is effective at reducing the spread of the pathogen. The practical challenge to this practice becomes the effective detection of colonized patients, with or without evident clinical symptoms. Exploring the effectiveness of cohorting under less ideal circumstances, with imperfect diagnostics, delays in diagnostic lab results, etc. remains an area for future work. Additionally, the contrast between Figs 5 and 6 versus Figs 3 and 4 suggest that this may only be true in circumstances where the admission rate of colonized patients is at or near zero. Even with a relatively low 7.79% admission prevalence, the benefit of cohorting is quickly swamped by the colonization pressure from these new colonized patients. It is possible that a more dynamic patient admission scheme (at present incoming patients are allocated randomly) might preserve the benefits of cohorting under some circumstances. The difficulties in implementing such a scheme on a routine basis for multiple pathogens in a clinical setting are considerable, and moreso in the case of emerging pathogens. Never the less, these results point to the considerable importance of an ICU’s population structure in shaping the dynamics of within-hospital infection transmission, highlighting the need for research into how these structures can be shaped by the hospital built environment, staff scheduling, hospital policy and other factors.
